# Editorial: Unraveling the interconnected dynamics of exercise metabolism, environmental stress, and nutritional factors

**DOI:** 10.3389/fphys.2025.1665703

**Published:** 2025-07-28

**Authors:** Ignacio Bartolomé Sánchez, Víctor Toro-Román, Jesús Siquier-Coll, Ciro José Brito

**Affiliations:** ^1^ Department of Physical Education and Sports, Pontifical University of Salamanca, Salamanca, Spain; ^2^ Faculty of Sports Sciences, University of Extremadura, Cáceres, Spain; ^3^ Department of Communication and Education, Universidad de Loyola, Sevilla, Spain; ^4^ Postgraduate Program of Physical Education, Federal University of Juiz de Fora, Juiz de Fora, Brazil

**Keywords:** exercise, metabolism, environment, nutrtion, stress

## Introduction

Human physiology operates within a complex triad: metabolic demands of physical exertion, environmental stressors, and nutritional inputs. This intricate interplay dictates performance, adaptation, and long-term health. Understanding these connections is vital for optimizing strategies in athletic training, clinical rehabilitation, and public health. This Research Topic explores how these factors converge, revealing novel insights into individualized interventions and mechanistic pathways. The contributions herein dissect hypoxia, fasting, and exercise modalities, collectively advancing our grasp of their synergistic or antagonistic roles.

## The hypoxia-exercise nexus: adaptation and individuality

Hypoxia, a potent environmental stressor, amplifies exercise-induced metabolic challenges but elicits highly variable responses. The study by Takei et al. on repeated sprint training in hypoxia (RSH) underscores this heterogeneity, revealing that peripheral oxygen saturation (SpO_2_) during training directly influences mechanical work and subsequent performance gains in sprinters. Crucially, nearly 20% of athletes derived no benefit, highlighting the need for personalized hypoxia prescriptions based on real-time physiological feedback. Complementing this, Jiang et al. demonstrate that during high-load resistance exercise under clamped hypoxia, severity matters: severe hypoxia (80% SpO_2_) amplified lactate, growth hormone, and epinephrine responses 30 min post-exercise compared to normoxia. Yet, the dominant stimulus remained the exercise itself—a reminder that environmental stress potentiates, but does not override, the primary metabolic signal.

Translating these insights to health applications, Tee et al. combined moderate hypoxia (FiO_2_ = 16.5%) with diverse exercise modalities in overweight adults. Sprint interval training (SIT) emerged as exceptionally efficient, improving post-exercise glucose regulation while being time-effective and well tolerated. This synergy of hypoxia and high-intensity exercise presents a practical strategy for metabolic health, particularly where time constraints limit traditional interventions. Further emphasizing hypoxia’s strategic utility, Xie et al. validated preacclimatization via intermittent hypoxia exposure (IHE) paired with exercise, to bolster tolerance to acute hypoxia. A 5-day program integrating moderate exercise with IHE elevated SpO_2_, reduced heart rate, and enhanced physical/mental performance at simulated 4,500 m. Notably, it also alleviated acute mountain sickness (AMS) symptoms, establishing combined protocols as superior to passive hypoxia exposure for altitude readiness.

## Nutritional modulation: fasting as a metabolic catalyst

Beyond environmental stressors, nutritional interventions like fasting recalibrate metabolic and signaling pathways. Juhas et al. investigated 8-day fasting and demonstrated profound shifts in kynurenine pathway metabolites, elevating neuroprotective compounds like kynurenic acid (KYNA) and xanthurenic acid (XA) at rest. Intriguingly, exercise post-fasting uniquely reduced metabolites linked to oxidative stress (3-hydroxyanthranilic acid), suggesting fasting may prime the body for exercise-induced redox regulation. These findings position fasting as a tool to amplify exercise’s health benefits through gut-brain-axis communication.

## Synthesis and future perspectives

Collectively, these studies illuminate three pillars: (a) Individualization is non-negotiable. Hypoxic interventions must account for SpO_2_ variability (Takei et al.) to avoid non-responsiveness. (b) Synergy drives efficacy. Combining stressors (e.g., hypoxia + SIT (Tee et al.) or fasting + exercise (Juhas et al.) yields superior outcomes versus isolated approaches. (c) Mechanistic specificity matters. Hypoxia’s effects are dose-dependent (Jiang et al.), while fasting distinctly reprograms tryptophan metabolism (Juhas et al.).

Future research should prioritize longitudinal studies to discern chronic adaptations, explore nutritional timing (e.g., fasting windows around exercise), and leverage omics technologies to map cross-talk between metabolic, immune, and neuroendocrine pathways. Additionally, expanding diversity in cohorts; particularly including female participants and clinical populations; will refine translational relevance. The work presented in this Research Topic transcends disciplinary silos, offering a scaffold for integrated approaches to human performance and health. As we unravel these dynamics, we move closer to precision interventions that harness environmental and nutritional stressors not as adversaries, but as allies in optimizing resilience.

